# Supplementary material for: Deep learning enables reference-free isotropic super-resolution for volumetric fluorescence microscopy
Source: Nat Commun. 2022 Jun 8;13:3297. doi: 10.1038/s41467-022-30949-6 (PMC9178036; doi:10.1038/s41467-022-30949-6)
Supplement: Supplementary file 1 — Supplementary Information [file 41467_2022_30949_MOESM1_ESM.pdf]

Supplementary Information

Deep learning enables reference-free isotropic super-resolution for volumetric fluorescence microscopy

Hyoungjun Park, Myeongsu Na, Bumju Kim, Soohyun Park, Ki Hean Kim, Sunghoe Chang, and Jong Chul Ye

Supplementary Figures

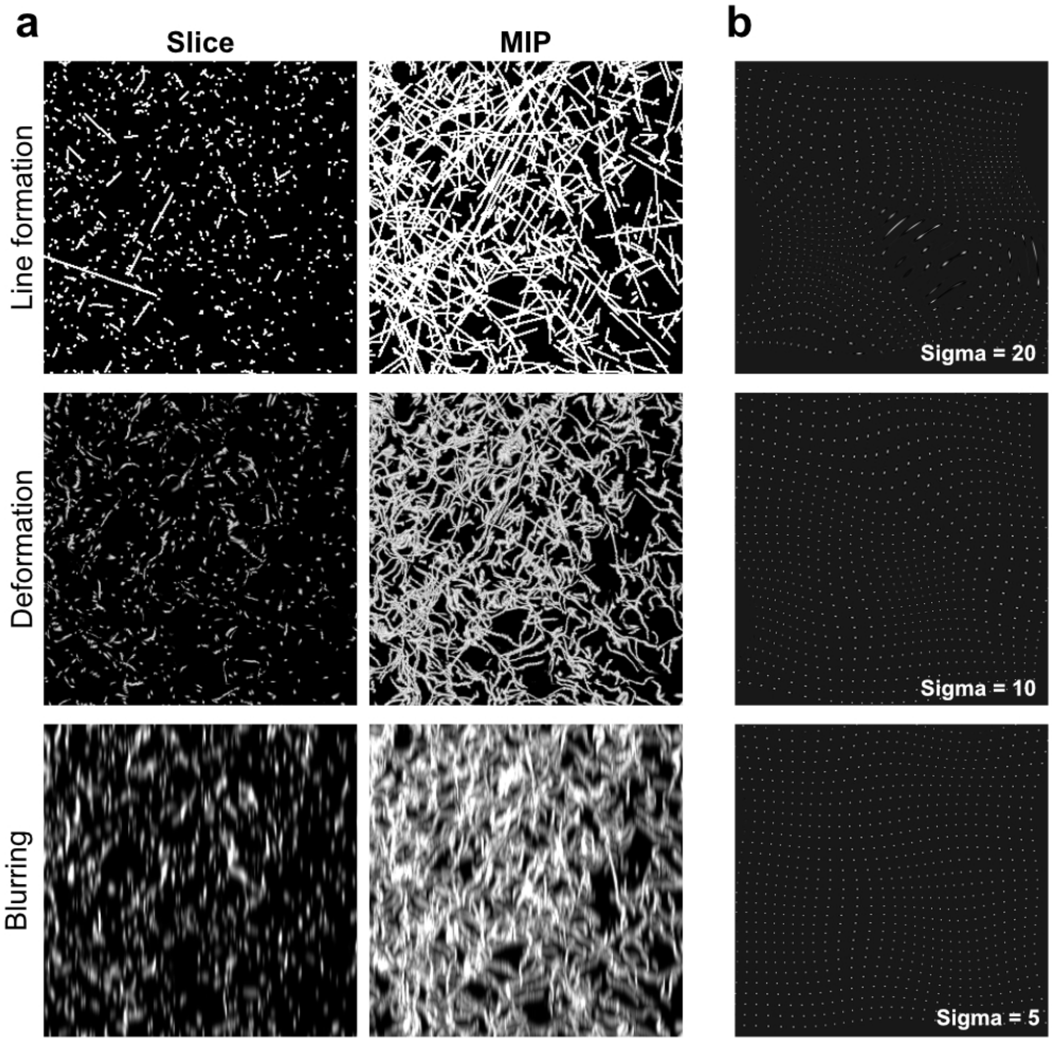

Supplementary Figure 1: Generation process of simulation data. a 10,000 linear lines of 2-pixel-thickness are drawn in the 700×700×700 voxel 3D space. For elastic deformation, we applied a grid-based deformation field with 70 grid locations with a sigma value of 3. For the blurring process, a Z-blurring Gaussian kernel is applied a standard deviation of 4. Maximum intensity projection images are of 30 slice thickness. **b** Examples of elastic grid-based deformation, with different degrees of deformation.

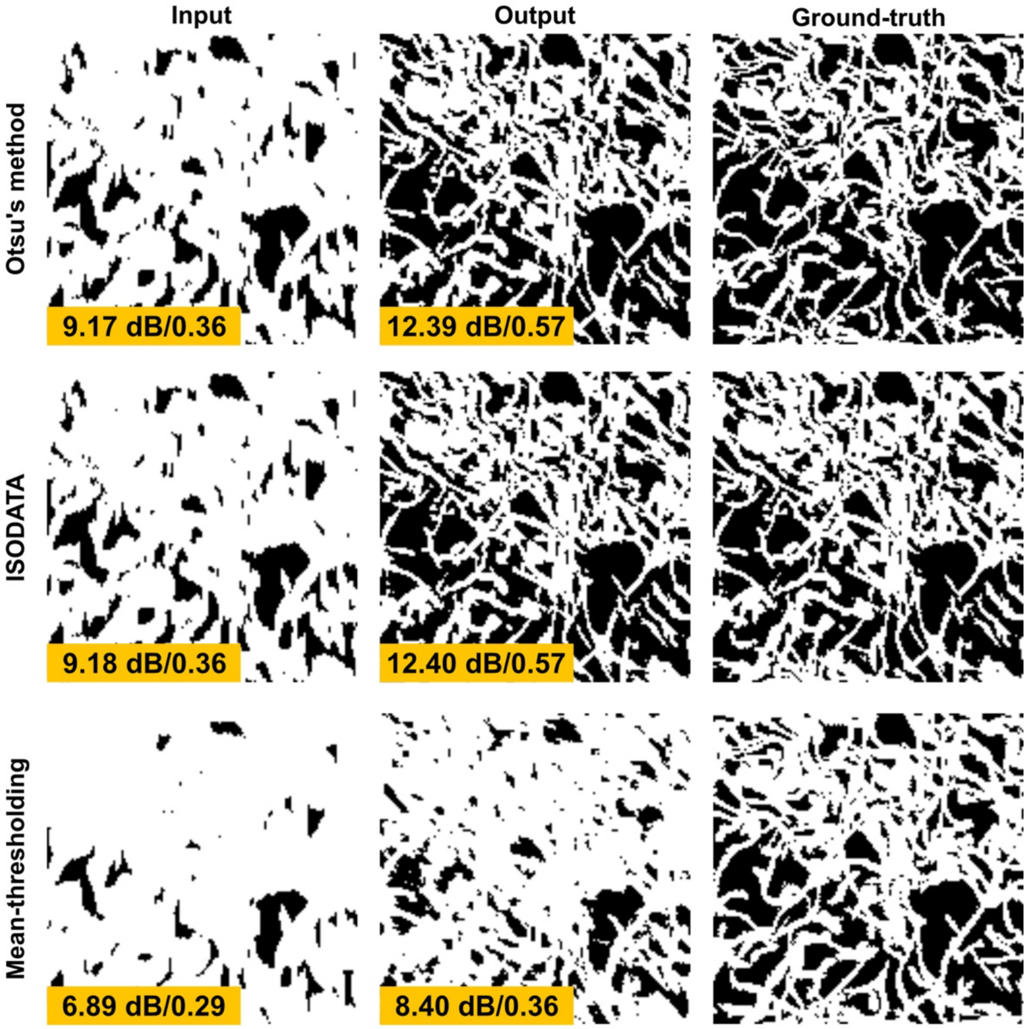

Supplementary Figure 2: Comparison of reconstruction performance across different segmentation methods. 2D ROI examples are shown for different binarization methods (Otsu's method, ISODATA, and mean-thresholding) with PSNR and SSIM metrics for the test volume of 700×700×700 voxels.

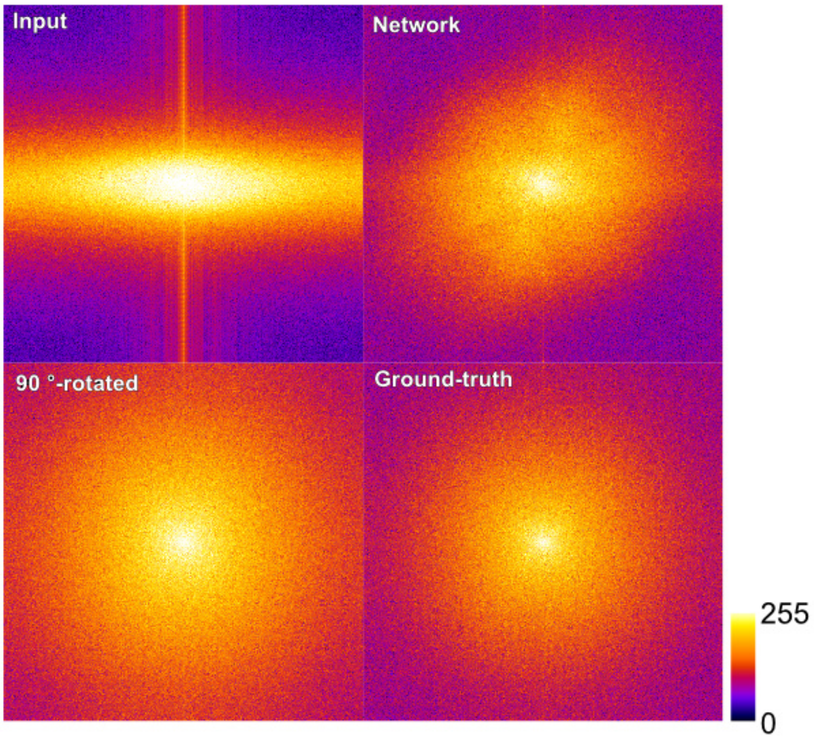

Supplementary Figure 3: Fourier spectrum analysis of synthetic axial projection images. The frequency profiles of the axial projection images of 15-slice-depth were generated using Fast Fourier Transform. The bilateral distribution of the frequency information in the input FFT image indicates the imbalance of the frequency information between x-axis and z-axis, along with loss of information shown as the vertical stripes. The frequency profile of the output image illustrates the restoration of the frequency information and also indicates approximation to the frequency information of the ground-truth and its lateral counterpart.

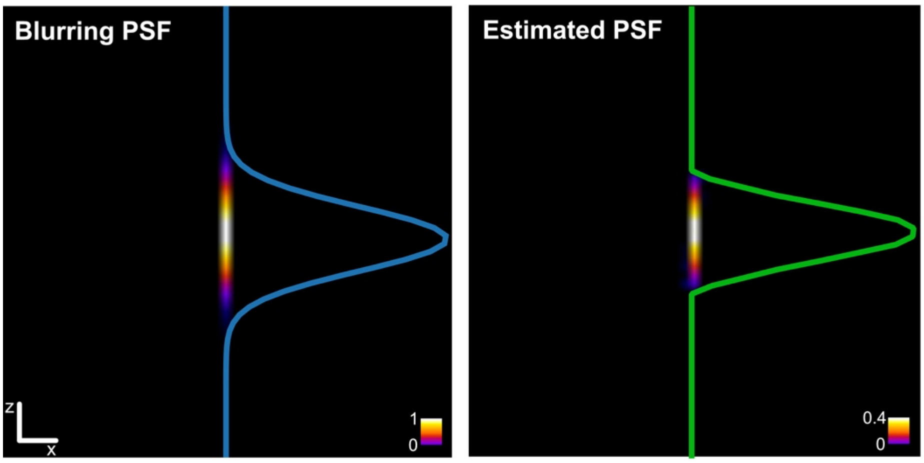

Supplementary Figure 4: Comparison of the blurring PSF and the PSF estimated as the impulse response to the generative network in the backward path.

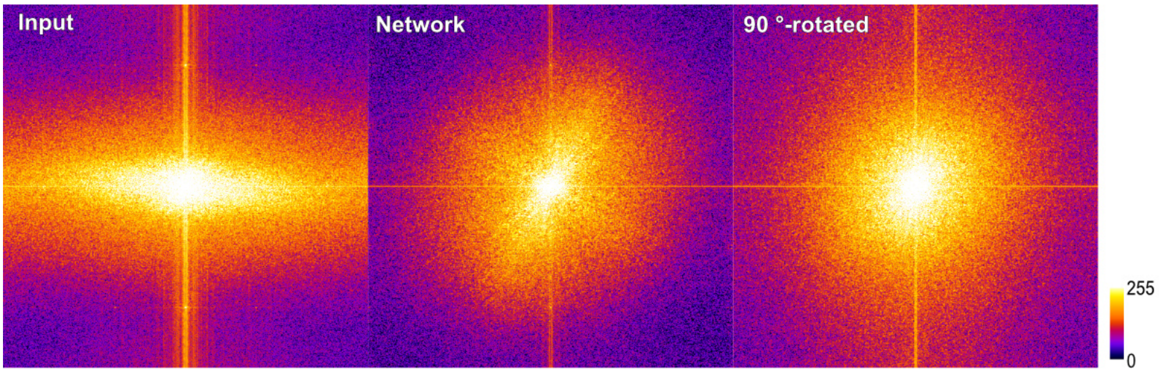

Supplementary Figure 5: Fourier spectrum analysis of the axial projection image from CFM. The top-ROIs in Fig. 2b, which span ~530×422μm^2, are used to using Fast Fourier Transform. The comparison is similar to that of Supplementary Fig 3 and indicates that the image degradation process in the CFM experiment is driven by the PSF elongation in the z-axis. The loss of information in the input axial image is shown as the bilateral distribution of the frequency information, while the frequency profile from the output exhibits restoration of such information as compared to that from the lateral imaging.

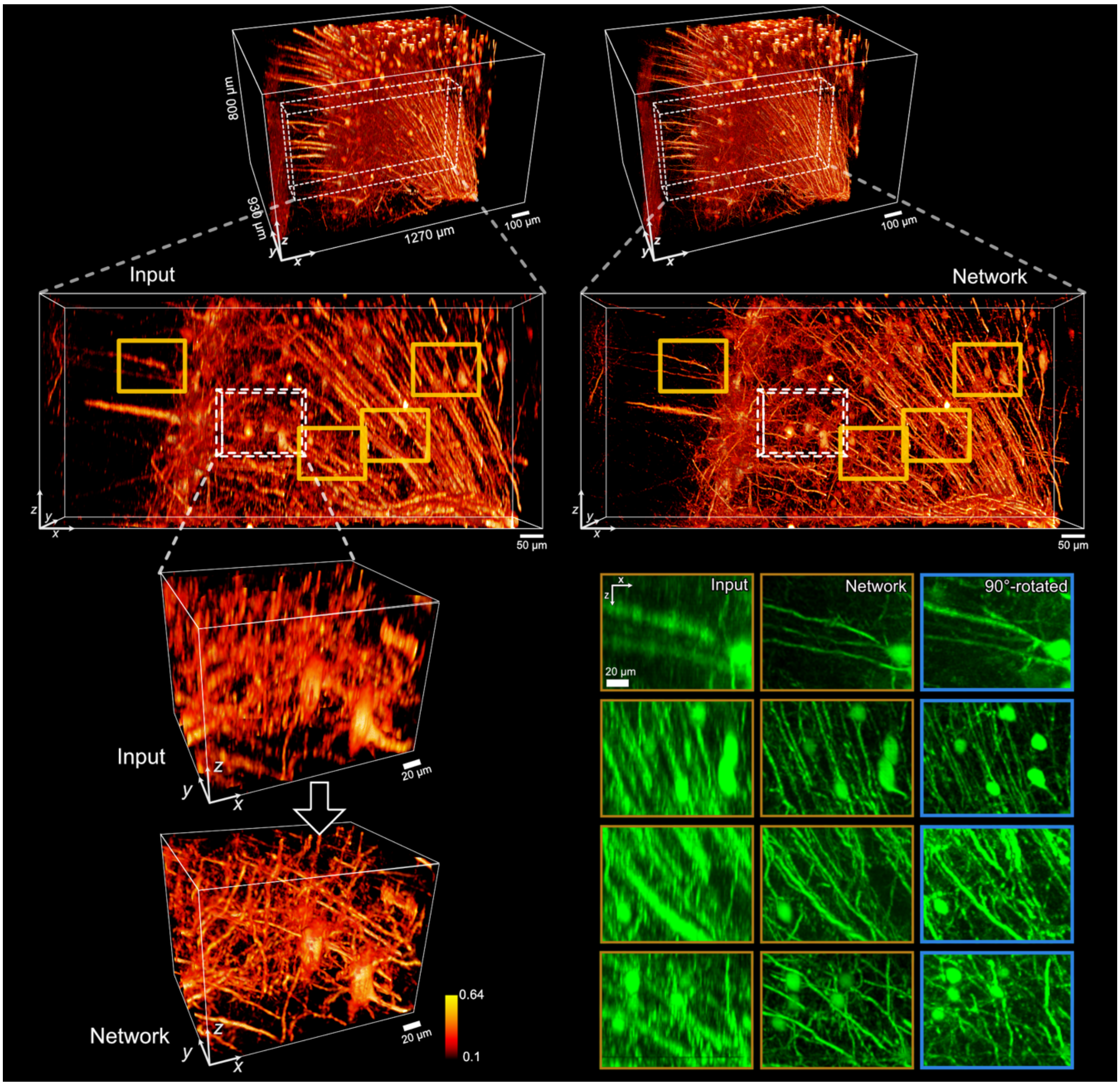

Supplementary Figure 6: Large-scale resolution improvement of the CFM image volume. 2D MIP images are from the yellow-box ROIs, in depths of 200 µm thickness. Experiments were repeated with four independently imaged volumes, achieving similar results. The color bar represents signal intensity normalized between 0 and 1.

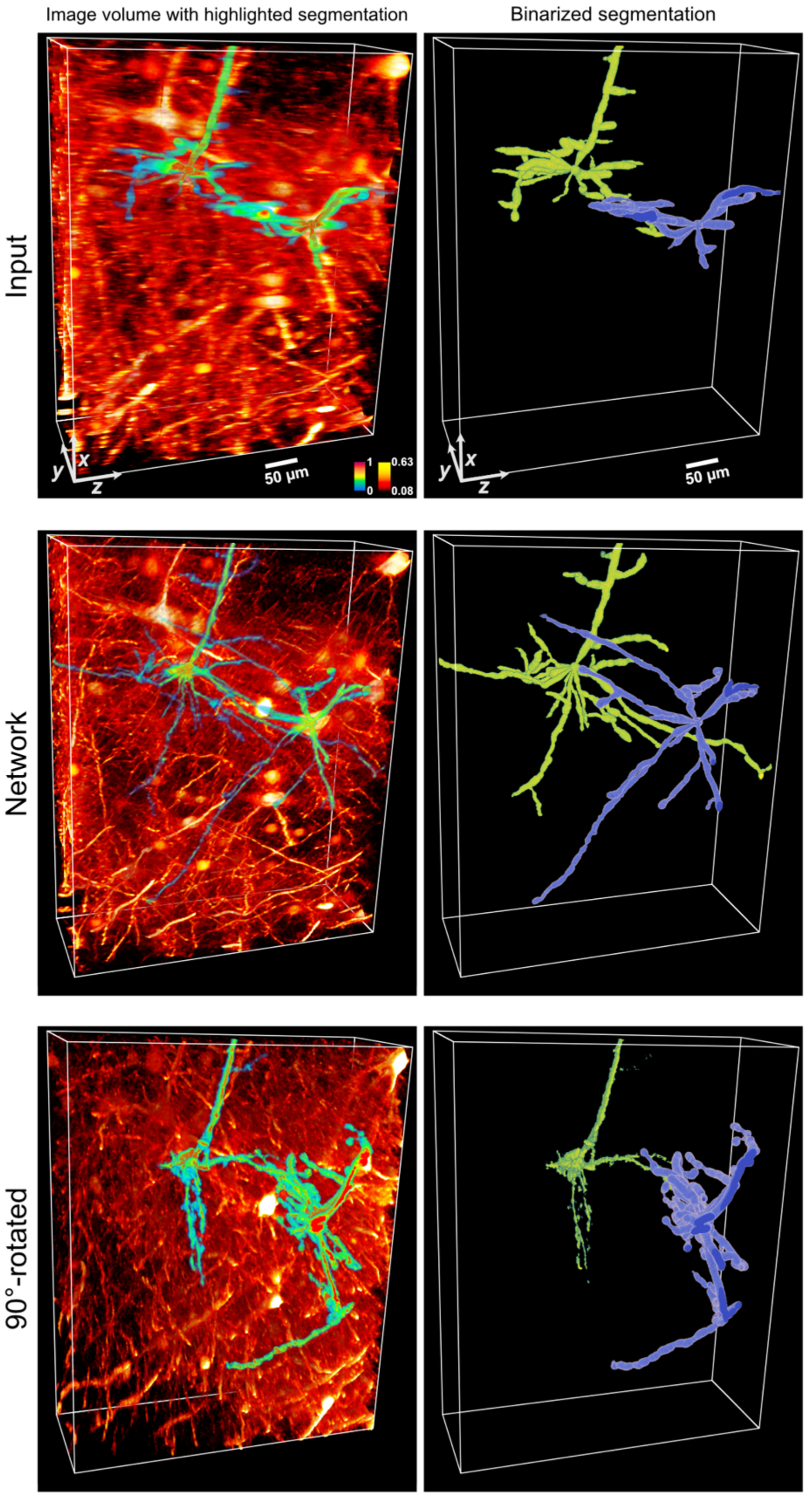

Supplementary Figure 7: Comparison of 3D traced reconstructions of two pyramidal neurons. The neuron tracings from both the input imaging and the 90°−rotated imaging were affected severely by anisotropy, while those in the output image volume form more extended structures regardless of the scanning direction. The color bars represent signal intensity normalized between 0 and 1.

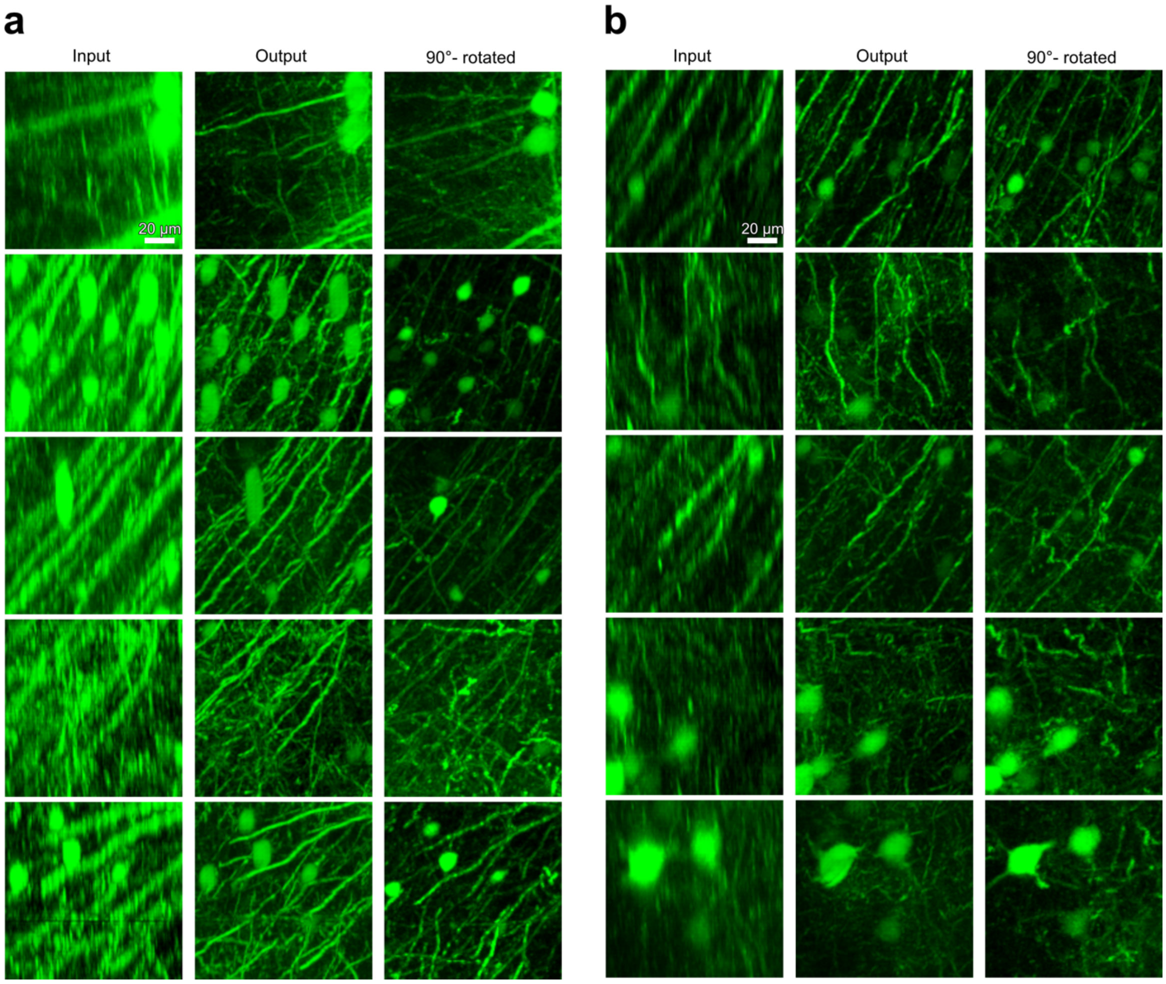

Supplementary Figure 8: ROI examples of 140×140 μm^2 for PSNR calculation. a ROI images with the five best PSNR metrics from the 31 ROIs for Fig. 2d. **b** ROI images with the five worst PSNR metrics from the 31 ROIs for Fig. 2d. As visually shown here, a signal-error-based metric such as PSNR did not necessarily reflect the resolution improvements. Images in both **a** and **b** are ranked from top.

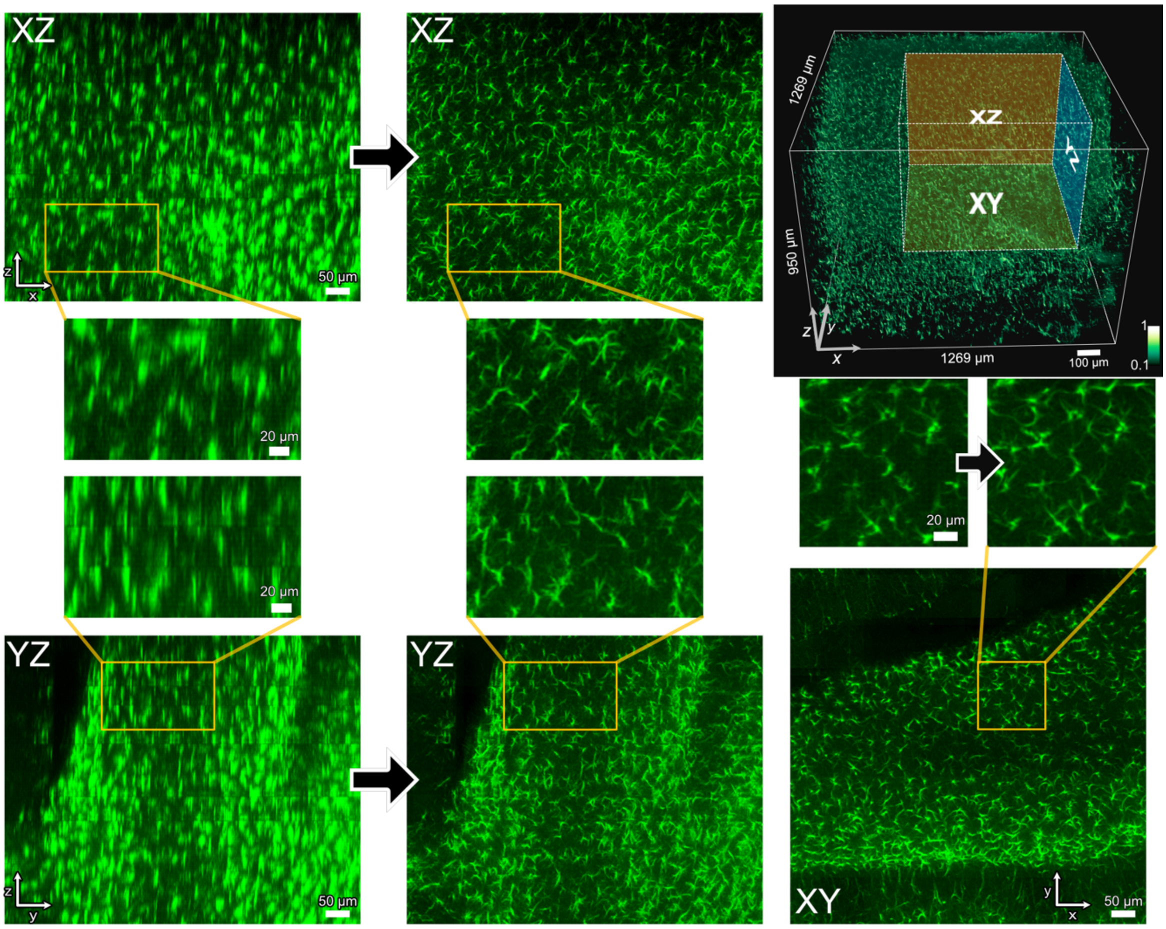

Supplementary Figure 9: Large-scale enhancement of axial resolution for astrocytes labeled with GFAP-antibody. A 1269×1269×950 μm^3 CFM image volume was used both for training and testing. The input-output relationship is marked by the arrow. The network output resolves the astrocytes in the axial plane, which were previously almost unidentifiable, while introducing no distortions in the XY plane. The overall image volume for training and testing is shown with ROIs for scale in the top right corner. 2D images are MIP images with thickness of 100 μm. The entire image space showed similar results. The color bar represents signal intensity normalized between 0 and 1.

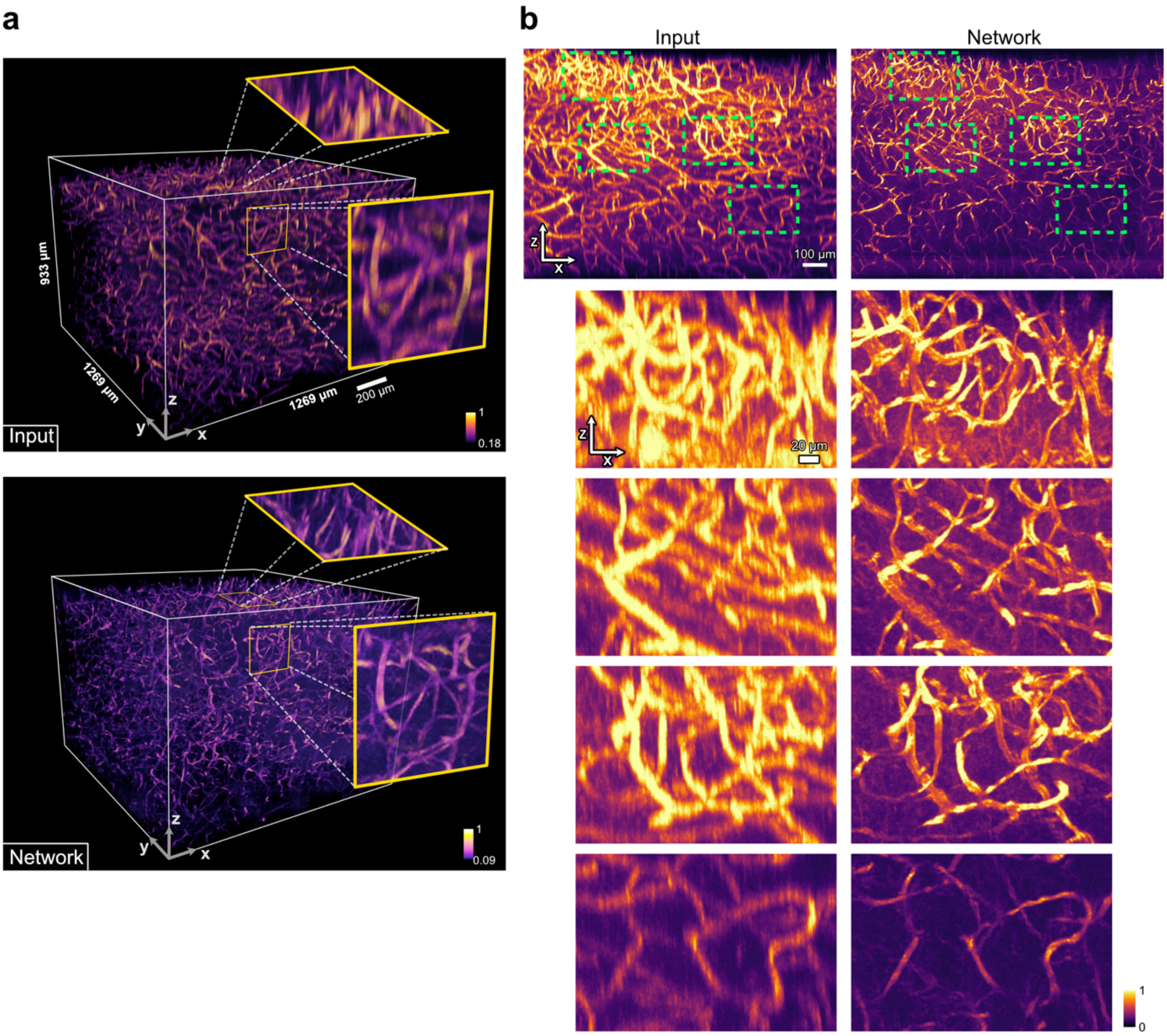

Supplementary Figure 10: Large-scale enhancement of axial resolution for blood vessels labeled with Lectin dye. a A 1269×1269×933 μm^3 CFM image volume was used both for training and testing. The resolution enhancement was global throughout the image space. The color bar represents signal intensity normalized between 0 and 1. **b** Axial MIP images in depths of 200µm before/after restoration with zoomed-in ROIs as green-dotted boxes. The entire image space showed similar results.

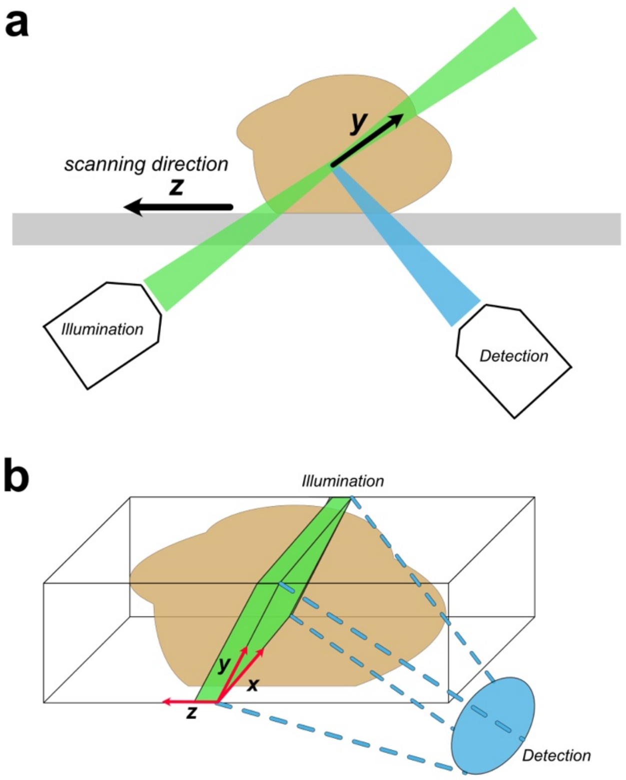

Supplementary Figure 11: Schematic of the OT-LSM system. **a** Lateral view of the imaging system. **b** 3D view of the image space with the imaging set-up.

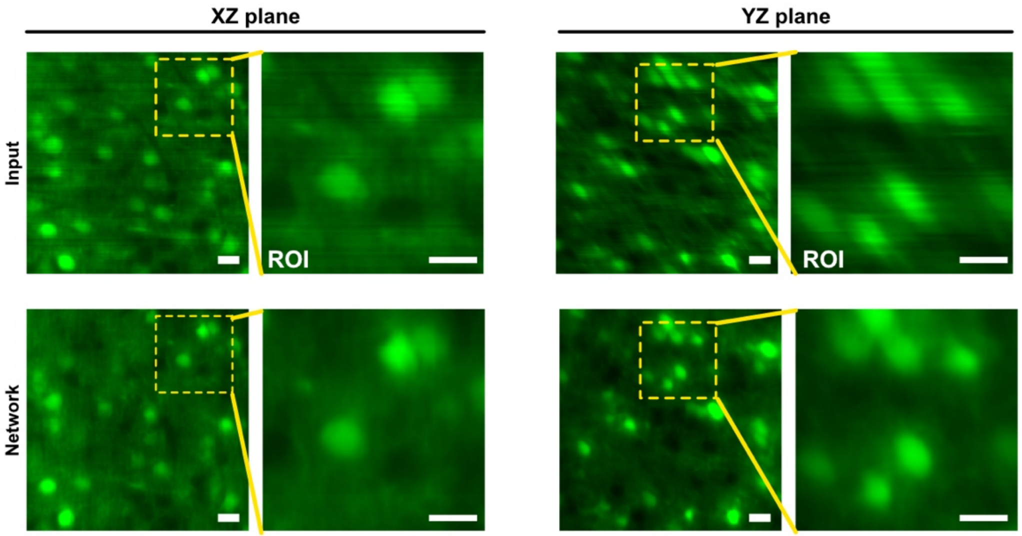

Supplementary Figure 12: Examples of the network removing imaging artifacts by stage drifts. Scale bars: 25 μm

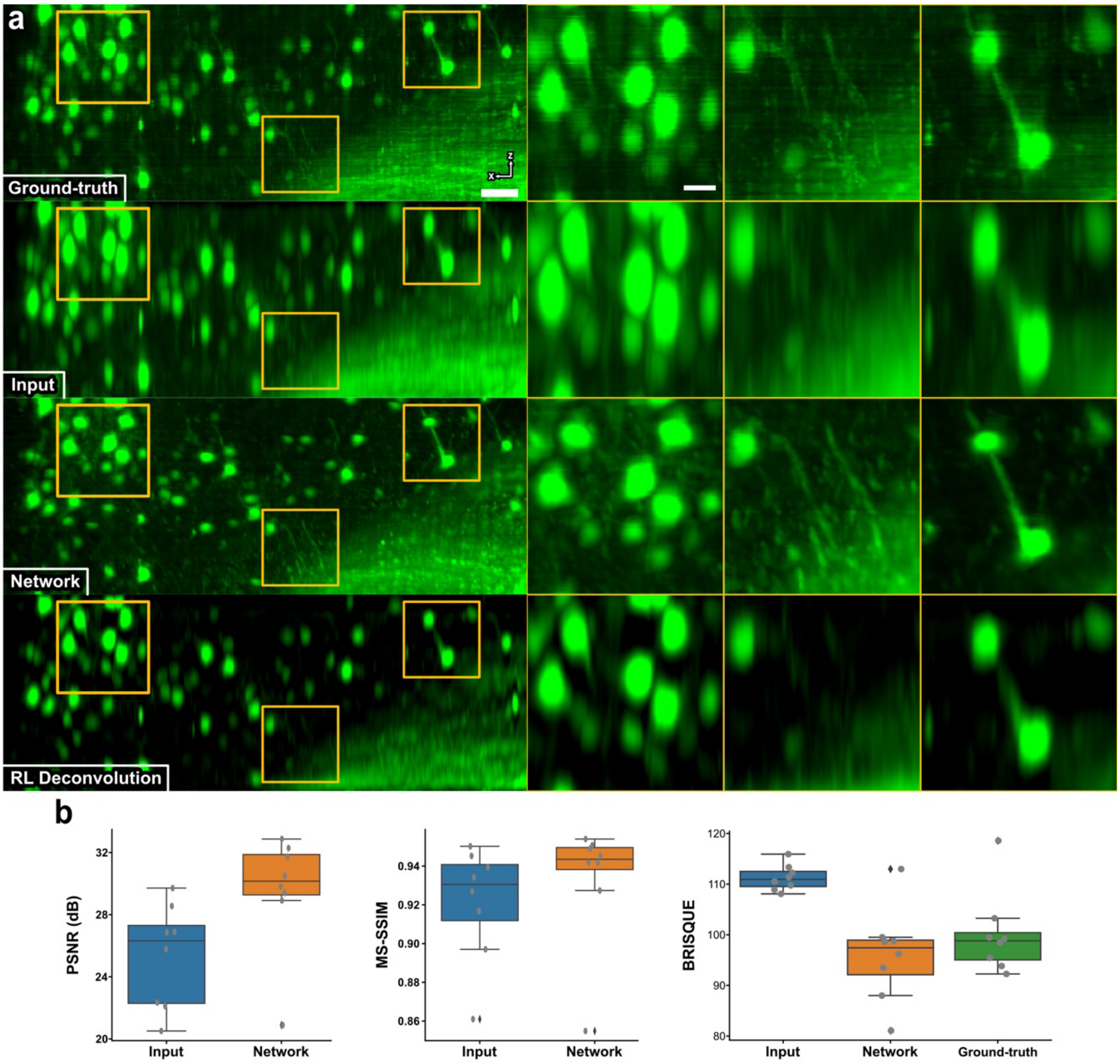

Supplementary Figure 13: Evaluation of the framework in the OT-LSM system, using a synthetically blurred image. a XZ-plane MIP images in depths of 130 µm. In comparison to the RL deconvolution image, the network not only deblurred the image but also reconstructed small details that are present in the high frequency information. **b** Quantification of resolution improvement. PSNR, MS-SSIM, and BRISQUE metrics were calculated for eight XZ-plane MIP images that are in depths of 17.5 µm (n=8 independent images). For BRISQUE score, a lower score indicates better perceptual quality. For the box plot, the box shows the IQR between Q1 and Q3 of the dataset, with the central mark showing the median and the whiskers indicating the minimum (Q1-1.5*IQR) and the maximum (Q3+1.5*IQR). Outliers are represented by diamond-shaped markers beyond the whiskers. Each dot represents one MIP image. Scale bars: 25 µm and 10 µm (Zoomed-in ROIs)

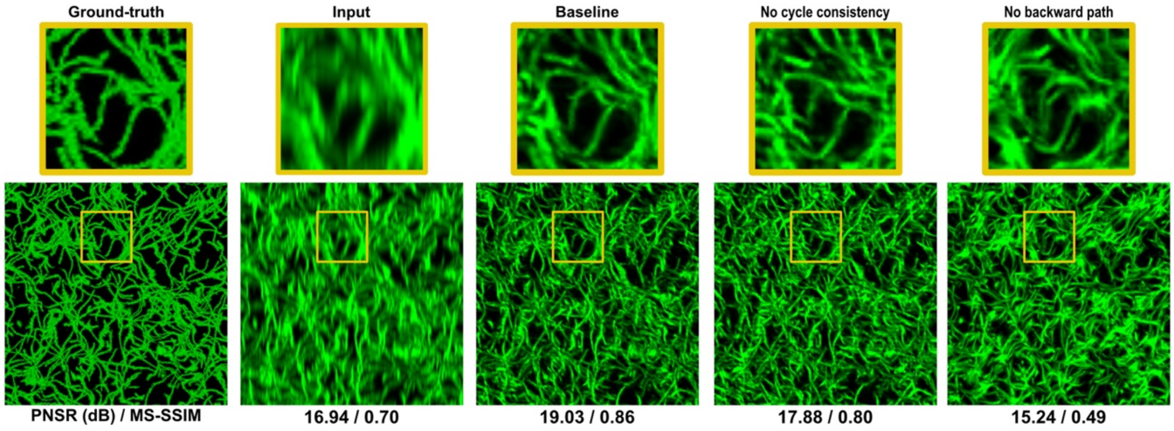

Supplementary Figure 14: Ablation study of the framework. The networks were trained on the synthetic image volume that was blurred with a Gaussian kernel with a standard deviation of 4. PSNRs and MS-SSIMs from the test volume are reported for quantitative comparison.

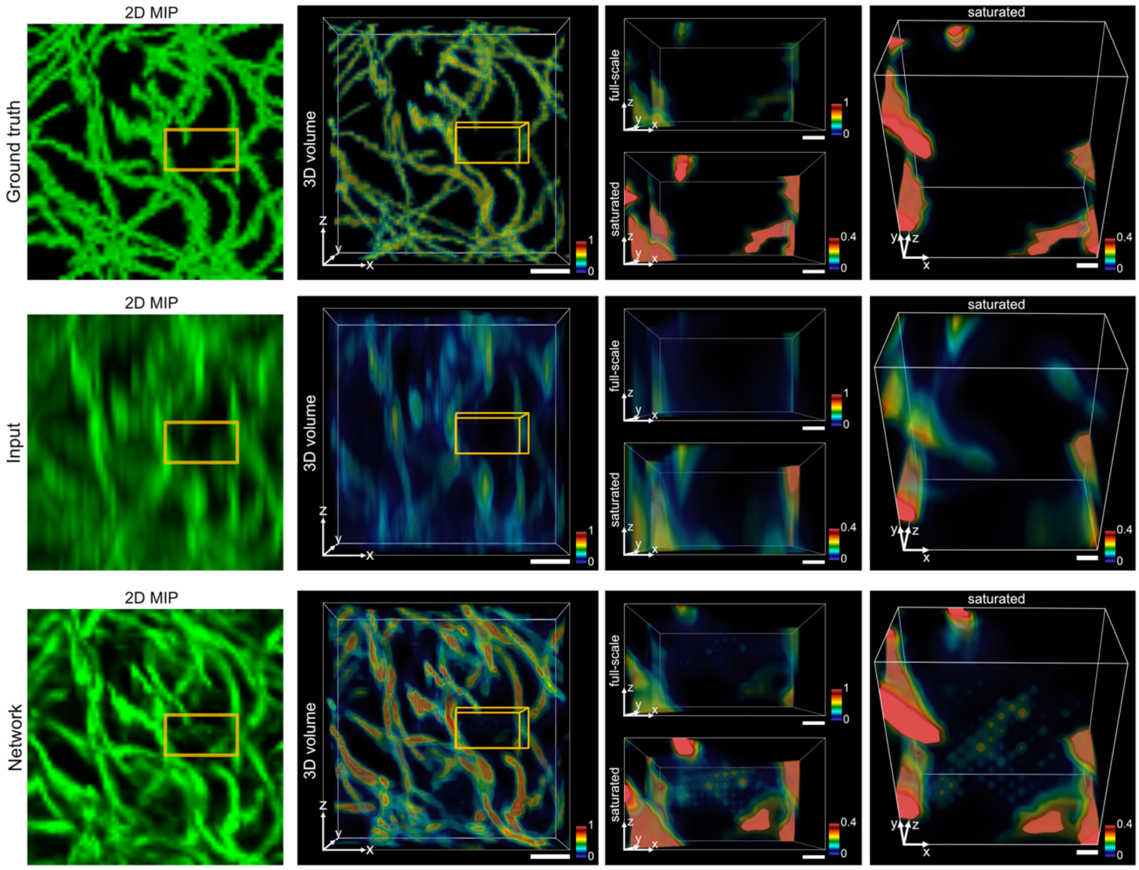

Supplementary Figure 15: Deeper look into possible hallucinations. We examined a MIP image of 20 slice thickness that contains a possible hallucination, which is labeled as the yellow box. 3D volumes were then visualized with zoomed-in ROIs on both the full intensity scale and the highly saturated scale (cut-off at 40%) to amplify the visibility of the possible artifact. In the 3D visualization, the possible artifact is not shown to be a spurious tubular structure. The color bars represent signal intensity normalized between 0 and 1. Scale bar: 10 voxels and 2 voxels (zoomed-in ROIs).

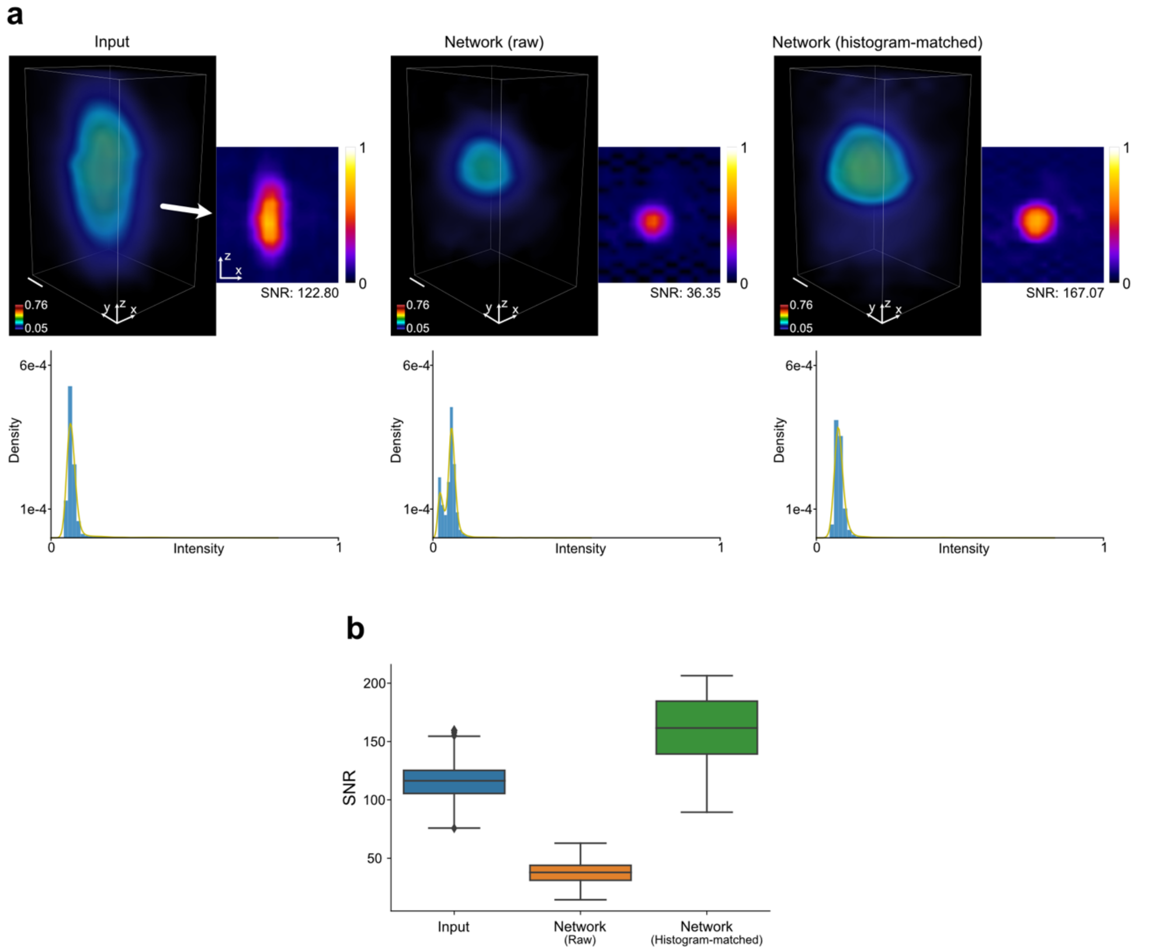

Supplementary Figure 16: Post-processing improves SNR. a Visualization of the ROI from Figure 3. as a single PSF model before/after image normalization as the post-processing step. The raw network output has an altered signal intensity distribution (histograms below. yellow-lines: Gaussian kernel estimates). This is primarily due to the one-volume training scheme that iterates through different sub-regions where the intensity distribution varies considerably: some sub-regions are void and some regions have clusters of bead objects. The SNR improves after normalizing to the histogram of the corresponding local input volume. The normalization is done via histogram matching[1]. The color bars represent signal intensity normalized between 0 and 1. **b** SNR distributions of fluorescent beads (n=305 spots from non-overlapping distinct regions). SNR is here defined as the ratio between the peak value of a ROI and the standard deviation of a background region (See Supplementary Note 7). The mean SNRs are 116.81±17.56 for input images, 37.91±9.29 for raw network output images, and 162.05±27.32 for normalized network output images. For the box plot, the box shows the IQR between Q1 and Q3 of the dataset, with the central mark showing the median and the whiskers indicating the minimum (Q1-1.5*IQR) and the maximum (Q3+1.5*IQR). Outliers are represented by diamond-shaped markers beyond the whiskers. Scale bar: **(a)** 1 µm.

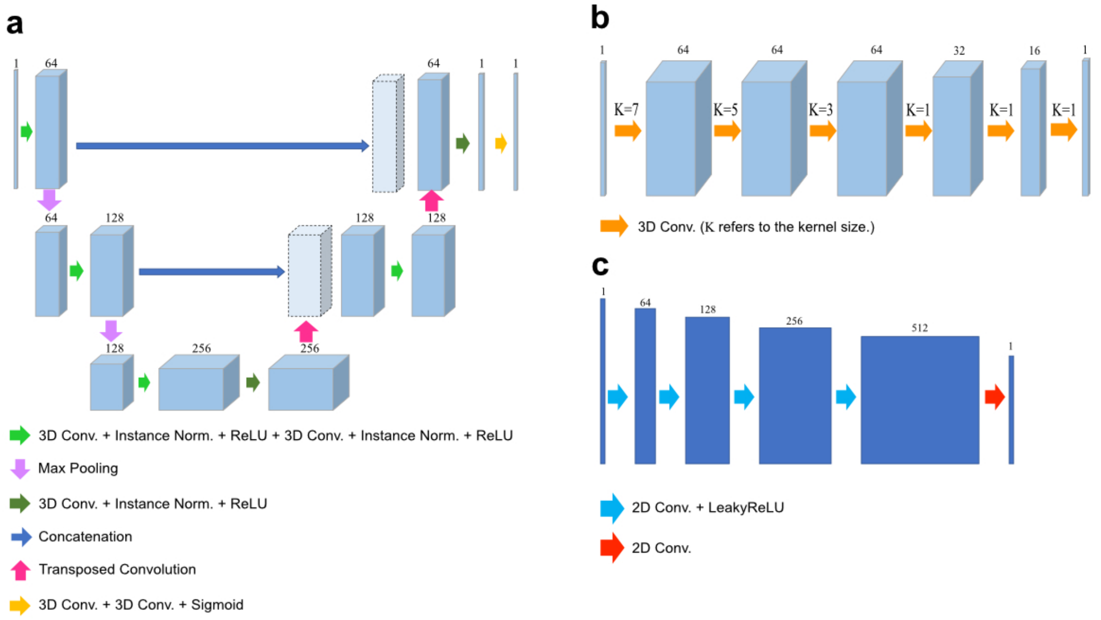

Supplementary Figure 17: Network designs for the generative networks and the discriminative networks. a 3D U-Net architecture for the generative network in the forward path. This architecture can be also used in the backward path. The kernel size for convolutions is set as 3. **b** 3D deep linear generator architecture for the generative network in the backward path. **c** 2D patch-GAN architecture for the discriminative network. The kernel size for convolutions is set as 4.

Supplementary Notes

Supplementary Note 1: Ablation studies

We additionally carried out two ablation studies on the synthetic image volume where one model excludes the cycle-consistency loss and the other excludes the backward path, which is of F and D_y. For fair comparison, we used the trained network models in the same condition, such as the training iteration. The results are shown in Supplementary Fig.14. In the first case, without the cycle-consistency, we noticed that while the network performance weakened in comparison to the baseline, the image was deblurred to some extent, shown by the improvement in the PSNR and MS-SSIM metrics. This is due to the design of the loss function still including the joint minimization of generation losses from both the blurring and deblurring. Nonetheless, as the metrics suggest, its reconstruction was not as accurate as the baseline, and this confirms that the cycle-consistency contributes to improving the reconstruction accuracy.

In the second case, without the backward path, we noticed that the generator still managed to generate convincing visuals, whose textures were comparable to those from the comparison models. This suggests that the lateral/axial sampling of the discriminative networks in the forward path is instrumental in generating refined details. However, without the backward path, the network cannot guide its learning to revert the image degradation process and hence ends up with inaccurate representations in the latent space modeling the high-resolution manifold. This is well reflected in the inaccurate reconstructions, as suggested by the exacerbated metrics of this ablation case.

Supplementary Note 2: Sample preparation for astrocyte and blood vessel CFM images

The animal experiments of Sparague-Dawley rats were performed according to the guideline of the Institute of Animal Care and Use Committee of Seoul National University (SNU-180321–7-4). For effective staining of a thick sample, the CLARITY clearing was performed before staining. First, anesthetized rats were transcardially perfused with 200 mL of cold phosphate-buffered saline (PBS), followed by 200 mL cold hydrogel monomer solution [4% acryamide (A8887, SIGMA), 0.25% 2,2-azobis [2-(2-imidazolin-2-yl) propane] dihydrochloride (VA-044) (225–02,111, Wako Pure chemical), and 4% paraformaldehyde (PFA, P6148, Sigma)] using peristaltic pump (MP-1000, Tokyo Rikakikai). The brains were harvested, then polymerized under de-gassed conditions at 37°C for 2-3 hours in an embedding device. And polymerized brains were sectioned with vibratome (VT1200s, Leica Biosystems, Germany) into 1-mm. The CLARITY clearing solution consisted of 4% (w/v) sodium dodecyl sulfate (SDS) (1610302, Bio-Rad), 50 mM lithium hydroxide (LiOH) (39225-0450, Junsei Chemical), and 25 mM boric acid (15663, SIGMA) in distilled water. CLARITY clearing was performed according to the original protocol[2,3] using Tissue Electrophoresis device (C phoresis, Crayon Technologies, Korea). Samples were placed in the holder, and 40 W of the constant electric power was applied. Then, cleared samples were washed in PBS for 2 days at 37 ℃. Immunostaining of cleared thick brain slices was carried out according to the previously published EFIC staining protocol[4]. The EFIC design combining the electric and magnetic force achieves a focused current flow into the sample, enabling rapid and efficient staining of the thick sample, while preserving the structural integrity. The sample incubated pretreat solution (20% dimethyl sulfoxide (D8418, SIGMA), 2% Tx-100 in PBS) and blocking solution (10% Bovine serum albumin (BSA) (Bovogen, Australia) in pretreat solution) for 1 hour then moved into holder of EFIC device (C stain, Crayon Technologies, Korea). GFAP-antibody (ab53554, Abcam) and DyLight 594 labeled Lycopersicon Esculentum (Tomato) Lectin dye (DL-1177, Vector Labs) were diluted (1:500) in staining buffer (5 % dimethyl sulfoxide (v/v), 2 % BSA (w/v), 25 mM boric acid and 50 mM LiOH in distilled water, pH 9.0). Then, staining buffer containing antibody was loaded, and 10 W of constant power was applied for 3 hours. After EFIC staining, electro-washing was performed for 15 minutes to remove un-bounded antibody. In the case of the GFAP staining, another round of the EFIC staining protocol was performed on the sample for staining with fluorescently conjugated secondary antibody (Alexa Fluor-488, A10055, Invitrogen,) (1:500 dilution in staining buffer). Before 3D-imaging using an upright confocal microscope (C2Si, Nikon), samples were incubated in RI matching solution for 1 hour. IMARIS 6.0 (Bitplane) software program were used for 3D visualization.

Supplementary Note 3: Generative network structure

Our generative network structure for the super-resolving path is based on the 3D U-Net architecture[5], as illustrated in Supplementary Fig. 17a. The generator is implemented as an encoder-to-decoder architecture and consists of the down-sampling path, the bottom layer, the up-sampling path, and the output layer. Specifically, the down-sampling path consists of the repetition of the following block:

$$f'_k = \text{ReLU}[\text{Norm}(\text{Conv}\{\text{ReLU}[\text{Norm}(\text{Conv}\{f_{k-1}\})]\})] \quad (1)$$
$$f_k = \text{MaxPool}[f'_k], \quad k = 1{,}2 \quad (2)$$

where f_k represents the output 3D feature tensor of the kth down-sampling block, and f_0 is the input 3D volume. ReLU[] is the rectified linear unit activation function with a slope of $\alpha = 1$, Norm() is the instance normalization[6], Conv{} is the convolution operation, and the Maxpool[] is the max pooling operation. The bottleneck layer is as follows:

$$g_0 = R\text{eLU}[\text{Conv}\{\text{ReLU}[\text{Conv}\{\text{ReLU}[\text{Conv}\{f_2\}]\}]\}] \quad (3)$$

where g_0 is the output of the bottom layer. The up-sampling path consists of the repetition of the following block:

$$g'_k = \text{ReLU}[\text{Norm}(\text{Conv}\{\text{ReLU}[\text{Norm}(\text{Conv}\{\text{Concat}[g_{k-1}, f'_{k-1}]\})]\})] \quad (4)$$
$$g_k = \text{TrConv}[g'_k], \quad k = 1{,}2 \quad (5)$$

where g_k is the output of the kth up-sampling block. Concat[] is the concatenation operation, and the TrConv{} is the transposed convolution. The last output layer is as follows:

$$y = \text{Sigmoid}[\text{Conv}\{\text{Conv}[g_2]\}] \quad (6)$$

where y is an output 3D volume.

The generative network architecture in the backward path is adjustable and replaceable based on how well the generative network can emulate the blurring or down-sampling process in the backward path. We searched for an optimal choice empirically between the 3D U-net architecture (refer to Supplementary Fig. 17a) and the deep linear generator without the down-sampling step (refer to Supplementary Figure 17b). The kernel sizes in the deep linear generator vary depending on depths of the convolution layers, as shown in Supplementary Figure 17b.

Supplementary Note 4: Discriminative network structure

As the inputs to the discriminator networks are XY, YZ, and XZ plane images, we adopted the discriminative network structure from 2D patchGAN[7] for our discriminator networks. The detailed schematic is illustrated in Supplementary Fig 17c. The patchGAN consists of multiple convolution blocks that allow the discriminator module to judge an input image based on different scales of patches.

$$v_k = \mathrm{LReLU}[\mathrm{Norm}(\mathrm{Conv}\{v_{k-1}\})], \quad \mathrm{k} = 1,2,3,4 \tag{7}$$

where v_0 is the input 2D image, either real or fake as generated by the generator network. Norm() is the instance normalization. LReLU[] is the leaky rectified linear unit activation function with a slope of $\alpha = 0.2$. The last layer is a convolution layer that generates a single channel prediction map.

Supplementary Note 5: Definition of PSNR

The PSNR metric in this study is calculated as follows:

$$\mathrm{PSNR} = 10 \log_{10} \left(\frac{N_x N_y \max\big(r(x,y)\big)^2}{\sum_{x=0}^{N_x-1} \sum_{x=0}^{N_y-1} [r(x,y) - t(x,y)]^2} \right) \tag{8}$$

where $r(x, y)$ is the reference image, $t(x, y)$ is the reconstructed image, and both images have the dimensions of N_x and N_y.

Supplementary Note 6: Definition of SSIM

The SSIM metric in this study is calculated on windows, x and y, as follows:

$$\mathrm{SSIM}(x,y) = \left(\frac{(2\mu_x\mu_y + c_1)(2\sigma_{xy} + c_2)}{(\mu_x^2 + \mu_y^2 + c_1)(\sigma_x^2 + \sigma_y^2 + c_2)}\right) \tag{9}$$

where x and y are two windows of pixel size $N \times N$. μ_x and μ_y are mean values of x and y, respectively, and σ_x^2 and σ_y^2 are variances. σ_{xy} refers to the covariance of x and y. c_1 and c_2 are set to stabilize the division with a weak dominator.

Supplementary Note 7: Definition of SNR

The SNR metric in this study is defined as follows[8]:

$$\mathrm{SNR} = \left(\frac{|s - \bar{b}|}{\delta_b}\right) \tag{10}$$

where s is the peak value of the signal calculated from a Gaussian fit to the particle, $\bar{b}$ is the mean value of a background ROI, and δ_b is the standard deviation of the background ROI. In our calculation, a background patch of $10 \times 10 \times 5$ voxels was used.

References

1. Gonzalez, R. C. & Woods, R. E. *Digital image processing* (Pearson, 2018).
2. Jensen, K., & Berg, R. W. Advances and perspectives in tissue clearing using CLARITY. *Journal of chemical neuroanatomy* **86**, 19–34 (2017).
3. Chung, K., Wallace, J., Kim, SY. et al. Structural and molecular interrogation of intact biological systems. *Nature* **497**, 332–337 (2013).
4. Na, M., Kim, K., Lim, H.R. et al. Rapid immunostaining method for three-dimensional volume imaging of biological tissues by magnetic force-induced focusing of the electric field. *Brain Struct Funct* **226**, 297–309 (2021)
5. Ronneberger, O., Fischer, P. & Brox, T. U-net: Convolutional networks for biomedical image segmentation. In Navab, N., Hornegger, J., Wells, W. M. & Frangi, A. F. (eds.) *Medical Image Computing and Computer-Assisted Intervention – MICCAI 2015*, 234–241 (Springer International Publishing, Cham, 2015).
6. Ulyanov, D., Vedaldi, A. & Lempitsky, V. Instance normalization: The missing ingredient for fast stylization. *Preprint at:* *https://doi.org/10.48550/arXiv.1607.08022* (2016).
7. Isola, P., Zhu, J.-Y., Zhou, T. & Efros, A. A. Image-to-image translation with conditional adversarial networks. *IEEE Conference on Computer Vision and Pattern Recognition (CVPR)*, 5967 (2017)
8. Wang, H. *et al.* Deep learning enables cross-modality super-resolution in fluorescence microscopy. *Nature Methods* 16, 103–110 (2019)
