# Supplementary material for: Deep learning enables reference-free isotropic super-resolution for volumetric fluorescence microscopy
Source: Nat Commun. 2022 Jun 8;13:3297. doi: 10.1038/s41467-022-30949-6 (PMC9178036; doi:10.1038/s41467-022-30949-6)
Supplement: Supplementary file 3 — Description of Additional Supplementary Files [file 41467_2022_30949_MOESM3_ESM.docx]

**Description of Additional Supplementary Files**

**Supplementary Movie 1**: **Verification of neuronal tracings.** Two examples of before/after verification of neuronal tracings from the network output image are shown. The tracings, which are marked as yellow contours, are overlaid on top of the reference image slices. In order to assess anatomical authenticity of the reconstruction, the tracings were manually verified in a slice-by-slice manner.

**Supplementary Movie** **2: Verification process for neuronal tracings.** The verification process for neuronal tracings from the network output image is illustrated. The tracings, which are marked as yellow contours, are overlaid on top of the reference image slices.  The preprocessing of the reference image is illustrated, along with examples of false-positives and true-positives.
