# Supplementary material for: Deep learning enables reference-free isotropic super-resolution for volumetric fluorescence microscopy
Source: Nat Commun. 2022 Jun 8;13:3297. doi: 10.1038/s41467-022-30949-6 (PMC9178036; doi:10.1038/s41467-022-30949-6)
Supplement: Supplementary file 6 — Reporting Summary [file 41467_2022_30949_MOESM6_ESM.pdf]

nature portfolio

Corresponding author(s): Jong Chul Ye
Last updated by author(s): 3/23/2022

Reporting Summary

Nature Portfolio wishes to improve the reproducibility of the work that we publish. This form provides structure for consistency and transparency in reporting. For further information on Nature Portfolio policies, see our Editorial Policies and the Editorial Policy Checklist.

Statistics

For all statistical analyses, confirm that the following items are present in the figure legend, table legend, main text, or Methods section.

n/a	Confirmed	
☐	☒	The exact sample size (*n*) for each experimental group/condition, given as a discrete number and unit of measurement
☐	☒	A statement on whether measurements were taken from distinct samples or whether the same sample was measured repeatedly
☒	☐	The statistical test(s) used AND whether they are one- or two-sided<br>*Only common tests should be described solely by name; describe more complex techniques in the Methods section.*
☒	☐	A description of all covariates tested
☒	☐	A description of any assumptions or corrections, such as tests of normality and adjustment for multiple comparisons
☐	☒	A full description of the statistical parameters including central tendency (e.g. means) or other basic estimates (e.g. regression coefficient) AND variation (e.g. standard deviation) or associated estimates of uncertainty (e.g. confidence intervals)
☒	☐	For null hypothesis testing, the test statistic (e.g. *F*, *t*, *r*) with confidence intervals, effect sizes, degrees of freedom and *P* value noted<br>*Give P values as exact values whenever suitable.*
☒	☐	For Bayesian analysis, information on the choice of priors and Markov chain Monte Carlo settings
☒	☐	For hierarchical and complex designs, identification of the appropriate level for tests and full reporting of outcomes
☒	☐	Estimates of effect sizes (e.g. Cohen's *d*, Pearson's *r*), indicating how they were calculated

Our web collection on statistics for biologists contains articles on many of the points above.

Software and code

Policy information about availability of computer code

Data collection	All data were collected using custom Python 3.8.11 codes that were written in-house. Pre-processing of data was done with custom Python codes that were written in-house and the Fiji software (Schindelin et al. , Nature Methods, 2012) with ImageJ version of 2.3.0.
Data analysis	Data augmentation, data normalization, and calculation of assessment metrics were performed using Python 3.8. The deep learning models were trained, validated and test using Python 3.8.11, and Pytorch 1.9.0. The code for deep learning models is available from the Zenodo repository: https://doi.org/10.5281/zenodo.6371391 or the Github repository: https://github.com/peterpark-git/neuroclear. Image registration was performed using BigWarp plug-in v7.0.4. Neuronal tracing was performed using NeuroGPS-Tree v1.0 (Quan et al., Nature Methods, 2015, https://doi.org/10.1038/nmeth.3662) and V3D v3.2. Image visualization was performed using the Fiji software with ImageJ v2.3.0. and Paraview v5.5.2.

For manuscripts utilizing custom algorithms or software that are central to the research but not yet described in published literature, software must be made available to editors and reviewers. We strongly encourage code deposition in a community repository (e.g. GitHub). See the Nature Portfolio guidelines for submitting code & software for further information.

Data

Policy information about availability of data

All manuscripts must include a data availability statement. This statement should provide the following information, where applicable:

- Accession codes, unique identifiers, or web links for publicly available datasets
- A description of any restrictions on data availability
- For clinical datasets or third party data, please ensure that the statement adheres to our policy

Training and test data for the simulation, the CFM experiment, the OT-LSM experiment for PSF deconvolution, and the OT-LSM experiment with artificial blurring

and test data for the OT-LSM experiment for artifact correction have been deposited in the Zenodo database under DOI:10.5281/zenodo.6352948. Training data for the OT-LSM experiment for artifact correction is available from the corresponding author upon reasonable request, due to size limitations. Source data are provided with this paper.

Field-specific reporting

Please select the one below that is the best fit for your research. If you are not sure, read the appropriate sections before making your selection.

- [x] Life sciences
- [ ] Behavioural & social sciences
- [ ] Ecological, evolutionary & environmental sciences

For a reference copy of the document with all sections, see nature.com/documents/nr-reporting-summary-flat.pdf

Life sciences study design

All studies must disclose on these points even when the disclosure is negative.

Sample size	Sample sizes are mentioned in the Results, Supplementary Fig. 6, 9 and 10. As the framework is reference-free and designed to be applicable to a image volume whose physical size is not particularly limited, no sample size calculation was performed explicitly; we consider the size of data to be sufficient since (1) the training process of the GAN-based architecture converges, (2) validation of the training performance suggests that the dataset was large enough.
Data exclusions	No data was excluded from the analyses.
Replication	Unless otherwise specified, all neural networks were trained once per set of hyper-parameters and input data. In terms of inference, all experiments were independently repeated at least three times per image volume, achieving similar results. Given that the hyper-parameters during the training and the inference setting, which include the step sizes, the over-lap sizes, and the border crop sizes during patch-by-patch inference of a trained model, were all kept consistent, all attempts at replication were successful. Reproducibility for biological replicates was tested and mentioned in Supplementary Figure 6, 9 and 10 (i.e. tested on biologically distinct samples).
Randomization	Group allocation into the validation set and the testing set for trained neural networks was randomized (i.e. separately randomized locations in the 3D image space).
Blinding	The data acquisition was done by N.M., B.K., and S.P. independently. Group allocation into the validation set and the testing set was done blindly to all the investigators. The data analysis was automatically performed and scored by computer algorithms; so blinding in data analysis was not necessary.

Reporting for specific materials, systems and methods

We require information from authors about some types of materials, experimental systems and methods used in many studies. Here, indicate whether each material, system or method listed is relevant to your study. If you are not sure if a list item applies to your research, read the appropriate section before selecting a response.

Materials & experimental systems

n/a	Involved in the study	
[ ]	[x]	Antibodies
[x]	[ ]	Eukaryotic cell lines
[x]	[ ]	Palaeontology and archaeology
[ ]	[x]	Animals and other organisms
[x]	[ ]	Human research participants
[x]	[ ]	Clinical data
[x]	[ ]	Dual use research of concern

Methods

n/a	Involved in the study	
[x]	[ ]	ChIP-seq
[x]	[ ]	Flow cytometry
[x]	[ ]	MRI-based neuroimaging

Antibodies

Antibodies used	Anti-GFAP (Abcam, ab53554) Lot#GR3221771-3, DyLight 594 labeled Lycopersicon Esculentum(Tomato) Lectin (Vector Labs, DL-1177) Lot#ZF-8026, Alexa Flour 488 Donkey anti-Goat IgG (H+L) (Invitroge, A10055) Lot#2211210

Validation	All antibodies were purchased from commercial manufacturers. Detailed validation statements can be found on the following manufacturer's websites.<br>1) Anti-GFAP: Species reactivity: Mouse, Rat, Human; Tested Application: WB (https://www.abcam.com/gfap-antibody-ab53554.html).<br>2) Alexa Fluor 488 Donkey anti-Goat IgG (H+L): Species reactivity: Goat ; Recommend Application: IHC, ICC, Flow (https://www.thermofisher.com/antibody/product/Donkey-anti-Goat-IgG-H-L-Cross-Adsorbed-Secondary-Antibody-Polyclonal/A-11055).<br>3) DyLight 594 labeled Lycopersicon Esculentum (Tomato) Lectin: Sugar specificity: [GlcNAc]1-3, N-Acetylglucosamine; Recommend Application: Immunofluorescence, Glycobiology (https://vectorlabs.com/dylight-594-labeled-lycopersicon-esculentum-tomato-lectin-lel-tl.html)

Animals and other organisms

Policy information about studies involving animals; ARRIVE guidelines recommended for reporting animal research

Laboratory animals	6-months-old male mice with genotyping including C57BL/6, Thy1-eYFP H-line and 3-weeks-old female Sparague-Dawley rats
Wild animals	No wild animals were used in this study.
Field-collected samples	No field-collected samples were used in this study.
Ethics oversight	Animal procedures for the mouse brain samples followed the animal care guidelines approved by the Institutional Animal Care Use Committee (IACUC) of the KBRI (IACUC-18-00018). Animal procedures for the rat brain samples followed the guideline of the Institute of Animal Care and Use Committee of Seoul National University (SNU-180321–7-4).

Note that full information on the approval of the study protocol must also be provided in the manuscript.
